# Biodegradable 3D-Printed Conjunctival Inserts for the Treatment of Dry Eyes

**DOI:** 10.3390/polym17050623

**Published:** 2025-02-26

**Authors:** Piyush Garg, Parvin Shokrollahi, Chau-Minh Phan, Lyndon Jones

**Affiliations:** 1 Centre for Ocular Research & Education (CORE), School of Optometry & Vision Science, University of Waterloo, 200 University Avenue West, Waterloo, ON N2L 3G1, Canada; p24garg@uwaterloo.ca (P.G.); p.shokrollahi@uwaterloo.ca (P.S.); chau-minh.phan@uwaterloo.ca (C.-M.P.); 2 Centre for Eye and Vision Research (CEVR), 17W Hong Kong Science Park, Hong Kong

**Keywords:** 3D printing, biodegradable polymers, conjunctival inserts, dry eye, matrix metalloproteinase-9

## Abstract

Purpose: To fabricate 3D-printed, biodegradable conjunctival gelatin methacrylate (GelMA) inserts that can release polyvinyl alcohol (PVA) when exposed to an ocular surface enzyme. Method: In this work, biodegradable conjunctival inserts were 3D-printed using a stereolithography-based technique. The release of PVA from these insert formulations (containing 10% GelMA and 5% PVA (P-Gel-5%)) was assessed along with different mathematical models of drug release. The biodegradation rates of these inserts were studied in the presence of a tear-film enzyme (matrix metalloproteinase-9; MMP9). The morphology of the inserts before and after enzymatic degradation was monitored using scanning electron microscopy. Results: The 3D-printed P-Gel-5% inserts formed a semi-interpenetrating network, which was mechanically stronger than GelMA inserts. The PVA release graphs demonstrate that at the end of 24 h, 222.7 ± 20.3 µg, 265.5 ± 27.1 µg, and 242.7 ± 30.4 µg of PVA were released when exposed to 25, 50, and 100 µg/mL of MMP9, respectively. The release profiles of the P-Gel-5% containing hydrogels in the presence of different concentrations of MMP9 showed the highest linearity with the Korsmeyer–Peppas model. The results suggest that the degradation rate over 24 h is a function of MMP9 enzyme concentration. Over 80% of P-Gel-5% inserts were degraded at the end of 8 h, 12 h, and 24 h in the presence of 100, 50, and 25 µg/mL MMP9 enzyme solutions, respectively. Conclusions: These results demonstrate the potential for 3D printing of GelMA for use as conjunctival inserts. These inserts could be used to deliver PVA, which is a well-known therapeutic agent for dry eye disease. PVA release is influenced by multiple mechanisms, including diffusion and enzymatic degradation, which is supported by morphological studies and biodegradation results.

## 1. Introduction

Dry eye disease (DED) is a multifaceted condition marked by tear film instability, inflammation, and sensory dysfunction. It is often associated with other eye health issues, including meibomian gland dysfunction, autoimmune disorders like Sjögren’s syndrome, and post-surgical effects from procedures such as LASIK. DED is categorized into aqueous-deficient and evaporative types, stemming from either insufficient tear production or excessive evaporation. Various factors, including environmental conditions, systemic diseases, medication use, and prolonged screen exposure, can worsen symptoms [1]. It is often characterized by high osmolarity of the tear film, with diagnostic threshold levels ranging from 305 to 316 mOsm/L [2], and inflammation of the ocular surface [3]. As the condition progresses, there is a noted escalation in both tear osmolarity levels and inter-eye variability, which is potentially useful in diagnosis and effective disease management [4,5]. This increased osmolarity initiates an inflammatory response, leading to cellular distress among epithelial cells and increased production of cytokines and matrix metalloproteinase-9 (MMP-9), an enzymatic protein involved in proteolysis [6]. Many studies have demonstrated elevated MMP-9 levels in the tears of individuals suffering from dry eye, which correlate closely with clinical examination findings [7,8,9]. The activity of MMP-9 increases with the severity of DED, contributing to compromised corneal epithelial barrier function, increased shedding of corneal epithelial cells, and irregularities in the corneal surface [10,11].

Eye drops are a common treatment for DED, but they have limited effectiveness and require frequent application. Other reasons contributing to the inefficiencies of the ophthalmic drops include low bioavailability, largely due to lacrimal secretion, rapid tear turnover, and efficient nasolacrimal drainage pathways. Thus, it would be helpful in the clinical management of DED to have a more efficient method to deliver products to the ocular surface that require less frequent dosing and remain on the ocular surface for an extended period.

An ideal ocular delivery system should possess properties such as biocompatibility, high drug residence time, low systemic absorption, and sustained drug release. Among the various strategies explored, integrating drug-loaded materials into ocular devices, such as contact lenses, shows the greatest potential for maximizing drug retention and bioavailability. Although contact lens-based drug delivery systems [12] can be used to prolong the ocular surface residence time, the burst release of drugs remains an unresolved issue [13,14,15]. Moreover, loading sufficient amounts of drugs and achieving drug concentrations at target sites equal to or above minimum effective levels remain a challenge [16]. To this end, conjunctival inserts, placed in the lower conjunctival sac beneath the lower eyelid, offer potential as alternatives to conventional ocular drug delivery systems. Depending on the typical daytime wearing period, the release needs to be within a few hours. Conjunctival inserts offer benefits such as precise dosing, reduced systemic absorption, minimal side effects, and sustained drug release. Recently, Duman et al. reported that a 3D-printed ocular insert based on polycaprolactone (PCL) combined with a liposomal strategy could support and enhance the delivery of moxifloxacin to the eye. The inserts were fabricated using a melt-extrusion 3D printing technique with a PCL filament. Moxifloxacin was loaded into liposomes, which were then incorporated into the PCL matrix during the printing process. A key finding of this work was the successful sustained release of moxifloxacin from the inserts over a period of 7 days in vitro. The ex vivo corneal permeation studies demonstrated significantly higher moxifloxacin penetration compared to conventional eye drops. The loading amount of moxifloxacin in the inserts was optimized, and the results showed a high encapsulation efficiency of the liposomal drug within the PCL matrix [17]. However, while supporting a relatively long-term release of Moxifloxacin for up to 7 days, the mechanical properties of PCL may compromise the patient’s comfort. In addition, PCL holds a slow biodegradation profile of up to two years, and, therefore, insert removal after the release period would be another concern. In another study, hot melt extrusion printing was coupled with fused deposition modeling for 3D-printing of a hydroxypropyl cellulose insert loaded with ciprofloxacin, and it was shown that the insert was capable of sustained ciprofloxacin release in vitro for over 24 h. The ex vivo studies using porcine corneas showed enhanced drug permeation compared to conventional eye drops [16]. Ocular inserts have been previously commercialized, and Ocusert™ was the first controlled-release polymer drug delivery system that exemplified this approach. Placed beneath the lower eyelid, this device administered the active ingredient pilocarpine to treat glaucoma. The device delivered the drug at a consistent rate over the course of one week, offering superior efficacy compared to pilocarpine eye drops, resulting in reduced incidence of side effects [18]. Ultimately, it was discontinued due to low uptake and the release of more effective drugs to treat glaucoma.

In addition, degradation is a necessity in biomedical applications [19]. Of note, biodegradable polymers have been used for fabricating drug delivery systems and are known to improve drug efficacy by controlling the release rate [20]. These polymers could also be molded in any shape or size and polymerized using photo-crosslinking. According to the International Union of Pure and Applied Chemistry (IUPAC), ‘degradable polymers’ undergo in vivo degradation resulting solely from hydrolysis by the water present in tissues and organs, referred to as hydrolysis or hydrolytic degradation [21]. Depending on the intended use, different products may require different stability or degradation properties [22]. For instance, in the case of chronic implantable biomedical devices, the polymers must be stable in biological environments for the device to perform its optimal function for a specified time period, which could last for a few years. Contrary to this, the degradation time for the polymers used to design tissue engineering scaffolds should be comparable to the tissue healing process, which could last from a few weeks to months. Similarly, the optimal degradation time frame for polymers used in drug delivery applications may range from a few hours to months. Several factors can control the rate of degradation of polymeric biomaterials, including crosslink density, degree of substitution, and the presence or absence of pendant groups. One such biodegradable material that could be used for this purpose is gelatin methacrylate (GelMA). GelMA is a biodegradable and biocompatible material used for a variety of biomedical applications and can be physically, chemically, or enzymatically crosslinked [23,24]. Of note, the photo crosslinking method used to make GelMA offers several advantages such as mild crosslinking conditions and low cytotoxicity [25]. Moreover, the biocompatible and biodegradable properties of GelMA could also make it possible to explore a new avenue to release incorporated drugs during passive exposures to various ocular enzymes [26].

Despite considerable advancements in the fabrication techniques of GelMA hydrogels and their extensive utilization across diverse biomedical disciplines, various challenges exist, such as control over pore size and distribution and reproducibility of mechanical properties, necessitating further investigation and development. Owing to the precision and high resolution required for the fabrication of biomedical devices from soft hydrogel-based biomaterials, in this work, a stereolithography (SLA)-based 3D-printing technique was employed to fabricate these conjunctival inserts from GelMA-based polymeric ink. Interestingly, SLA-based 3D printing makes it possible to use different photopolymerizable materials loaded with therapeutics of interest [27]. One such therapeutic molecule of interest is polyvinyl alcohol (PVA), which is a commonly used viscosity enhancer in eye drops [28], with the capability of reducing symptoms of dry eye by stabilizing the tear film and reducing evaporation. Many ocular lubricants to treat moderate dry eye contain PVA [29].

In this work, we planned to combine the favorable properties of GelMA and PVA to design prototype biodegradable conjunctival inserts, which could potentially be used for the treatment of dry eyes. Interestingly, GelMA degrades in the presence of collagenases or MMPs, and these enzymes are also found on the ocular surface. Additionally, in various ocular conditions, including moderate to severe dry eyes, the levels of MMP9 enzymes are elevated [7,30]. The MMP9 enzyme cleaves collagen/gelatin chains [31,32], thereby providing a stimulus for the enzymatic degradation of GelMA. As the GelMA chains are cleaved, the PVA molecules from the network structure are released, helping to protect the ocular surface against the negative impact of the DED process.

## 2. Materials and Methods

### 2.1. Materials

Type A gelatin (gel strength 300), methacrylic anhydride, sodium carbonate, sodium bicarbonate, acetic acid, tartrazine, lithium phenyl-2,4,6-trimethylbenzoylphosphinate (LAP), and PVA (molecular weight 89–98 kDa, 99+% hydrolyzed) were all purchased from Sigma, St. Louis, MO, USA. Phosphate buffered saline (PBS) was purchased from Fisher Scientific, Waltham, MA, USA. All chemicals were used without further purification unless mentioned otherwise. Water for all the reactions, solution preparation, and polymer purification was ultrapure.

### 2.2. Synthesis of GelMA

GelMA was synthesized by reacting 40 g of Type A gelatin with methacrylic anhydride (1% *v*/*v*, Sigma, St. Louis, MO, USA) in 400 mL of carbonate-bicarbonate buffer (12.5 mM sodium bicarbonate and 87.5 mM sodium carbonate, pH 9.4) at 50 °C for 1 h. To terminate the reaction, 6 mL of 10% acetic acid was added. The product was then dialyzed using 12–14 kDa dialysis membranes (Sigma, St. Louis, MO, USA) in deionized water for 24 h to eliminate low molecular weight impurities. After dialysis, the GelMA solution was freeze-dried and stored at −80 °C for future use.

### 2.3. Preparation GelMA and P-Gel-5% Ink

To prepare the P-Gel-5% prepolymer solution, GelMA (20% *w*/*v*) was dissolved in LAP solution (1.2% *w*/*v*, Sigma 900889, St. Louis, MO, USA) in phosphate-buffered saline (PBS, Fisher Scientific, Waltham, MA, USA). Separately, a PVA solution (10% *w*/*v*, molecular weight 89–98 kDa, 99+% hydrolyzed, Sigma, St. Louis, MO, USA) was prepared in PBS and heated at 90 °C until fully dissolved. The two solutions were combined in a 1:1 ratio, resulting in the P-Gel-5% prepolymer solution containing GelMA (10% *w*/*v*), PVA (5% *w*/*v*), and LAP (0.6% *w*/*v*). For the preparation of GelMA ink, GelMA (10% *w*/*v*) was dissolved in LAP solution (0.6% *w*/*v*) made in PBS at 37 °C. Tartrazine was added (photo-absorber, 0.3% *w*/*v*) [33] to GelMA and P-Gel-5% inks and incubated at 37 °C for 1 h prior to use (Table 1).

### 2.4. 3D Printing of P-Gel-5% Conjunctival Inserts

**Figure 1:**
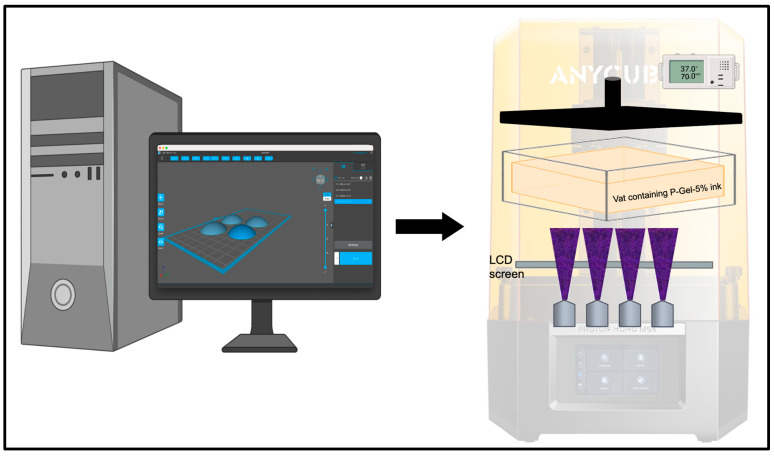
Fabrication of GelMA and P-Gel-5% conjunctival inserts using 3D printing.

Ocular inserts of 4 mm diameter and 1000 µm thickness were designed using computer-aided design software (FreeCAD 0.19) and then 3D-printed using a commercial masked-stereolithography (mSLA) 3D printer (AnyCubic Photon Mono M5, Shenzhen, China). The printer was retrofitted with a humidity and temperature control kit to enable printing at ~95% humidity and 37 °C (Figure 1). The GelMA and P2-Gel-5% prepolymer ink solutions were mixed gently before use. The 3D-printing photopolymerization process was performed with UV light of wavelength 405 nm. The overall thickness of the insert was 1000 µm, and they were printed with a layer thickness of 100 µm and exposure time of 120 s [33].

### 2.5. Mechanical Testing

Mechanical properties of the GelMA and P-Gel-5% hydrogel samples were measured in compression mode using a universal testing machine (UTM, Texture Analyser XT Plus, Hamilton, MA, USA). The test was conducted on hydrogel samples (12 mm height and 10 mm diameter) that were swollen for 24 h in PBS buffer at pH 7.4. The samples were gently dabbed and placed in the middle of the stage, and a load cell of 5 kg was applied. Each measurement was repeated three times.

### 2.6. Equilibrium Swelling

Swelling ratios of the hydrogels were determined using a gravimetric approach. Initially, the hydrogel discs were freeze-dried (at −80 °C and 133 × 10^−3^ mbar), and their weights were recorded. They were then immersed in 4 mL of PBS at 35 °C with gentle agitation at 50 rpm, allowing them to swell for 24 h. At specific time intervals, the samples were taken out of the PBS; excess surface water was carefully blotted off, and their weights were measured at specific time intervals until equilibrium swelling was achieved. The swelling ratio at any given time *t* was calculated using the following equation:

Swelling ratio = (W*_t_* − W*_i_*)/W*_i_* × 100

where W*_i_* is the initial dry weight of the hydrogels, and W*_t_* is the weight of the swollen hydrogels at time *t*.

### 2.7. Water Contact Angle

To assess the hydrophilicity of the hydrogels, water contact angles of the GelMA and P-Gel-5% samples were measured using an Optical Contact Analyzer (DataPhysics Instruments GmbH, Filderstadt, Germany) with the sessile drop technique. Briefly, an 8 μL droplet of high-performance liquid chromatography (HPLC) grade water was dispensed onto the hydrogel surface at a rate of 2 μL/s and imaged after the droplet stabilized. The water contact angles from the captured images were analyzed using SCA 20 software (version 2.04, Build 4). For each hydrogel, measurements were taken from three separate droplets across the surface, and the final values represent the average of these independent measurements.

### 2.8. Biodegradation of P-Gel-5% Inserts by MMP9

P-Gel-5% inserts (4 mm diameter and 1000 µm thickness) were 3D-printed and briefly dried using a Kimwipe^TM^ (Kimberly-Clark Professional 34155, Irving, TX, USA), and their weights were measured on a scale and recorded. Next, inserts were added to 1.5 mL microcentrifuge tubes with 1 mL of enzyme solution containing varying MMP9 (Gibco, Billings, MT, USA) concentrations dissolved in 1X PBS. Samples were incubated, with gentle agitation, at 37 °C. At each indicated time point, samples were briefly vortexed, and 50 µL of the solution was removed for detection of PVA.

### 2.9. Scanning Electron Microscopy

To investigate the biodegradation mechanism of the conjunctival inserts, the 3D-printed samples were incubated in a 50 µg/mL MMP9 enzyme solution for 24 h. Following degradation, the inserts were frozen at −80 °C overnight and subsequently freeze-dried for 24 h using a Labconco Freeze Dry System Freezone 2.5 Liter (7670000, Kansas City, MO, USA). The freeze-dried samples were then mounted on an aluminum stub using double-sided adhesive conductive carbon tape and analyzed using environmental scanning electron microscopy (ESEM, Quanta FEG 250, FEI Company, Hillsboro, OR, USA).

### 2.10. Release and Detection of PVA

The ocular inserts containing 5% (*w*/*v*) PVA (P-Gel-5%) were incubated in glass vials with 1 mL of PBS solution at 37 °C and 50 rpm. At each indicated time point, 200 µL of the solution was removed for detection of PVA and replaced with fresh PBS. For detection of PVA, a solution of iodine (150 mM potassium iodide, 50 mM diiodine in deionized water, Sigma 221945 and 207772, St. Louis, MO, USA) and borate (64.7 mM boric acid in deionized water, Sigma B0394, St. Louis, MO, USA) was mixed in 1:5 ratio of iodine solution:borate solution to generate the PVA detection solution. The solution was gently inverted 4–6 times in a conical tube to ensure even mixing. To measure PVA in solution, 163.6 µL of PVA detection solution was added to 36.4 µL of samples of interest in a 96-well plate and incubated at room temperature for 20 min with gentle shaking. The absorbance of the unknown samples, in addition to a set of standard samples of known PVA concentrations prepared in 1X PBS, was read at 630 nm using an Imaging Multimode Plate Reader (Cytation 5, Agilent BioTek Instruments, Winooski, VT, USA) [34]. The PVA release was quantified using a calibration curve method. This curve was plotted for standard solutions ranging from 10–250 µg/mL. The *R*^2^ value for the calibration curve was 0.9997.

### 2.11. Drug Release Kinetics via Mathematical Modeling

To evaluate the release kinetic profiles of the samples over the studied time duration, different mathematical models were studied [35]. These included the zero- and first-order release model, Higuchi release model, and Korsmeyer–Peppas model. The graphs were plotted as mentioned in Table 2, and the coefficient of determination (*R*^2^) was calculated for each model [36].

### 2.12. Statistical Analysis

Statistical analysis was performed using GraphPad Prism Version 10.0 (GraphPad Software, La Jolla, CA, USA). All data are reported as mean and standard deviation unless otherwise stated. Two-way ANOVA was performed to determine differences across testing groups wherever applicable. Tukey’s multiple comparison tests were used when necessary. In all cases, statistical significance was considered significant for a *p*-value of <0.05 unless otherwise stated.

## 3. Results and Discussion

### 3.1. The 3D Printing of Ocular Inserts

**Figure 2:**
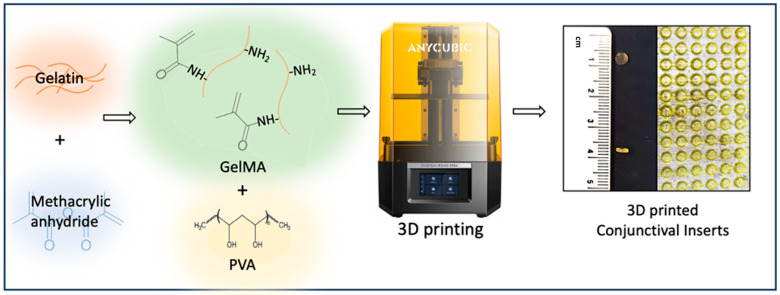
3D printing of PVA-loaded conjunctival inserts.

Figure 2 shows the 3D-printed P-Gel-5% conjunctival inserts. The discs were 4 mm in diameter and 1000 μm in height and were printed in about 30 min. Multiple conjunctival inserts could be fabricated at once, as shown on the print plate of the 3D printer. Notably, 3D printing offers precise control over dimensions, ensuring reproducibility. Previous studies indicate that a good photocurable biomaterial ink for projection-based printing techniques such as SLA LCD printing should meet three major requirements: (a) rapid photo-crosslinking rate, (b) dense crosslinking structure, and (c) excellent fluidity (for rapid filling of gaps and spaces) [37]. In this work, GelMA was selected as a base material owing to its biocompatibility and demonstrated high levels of controllability as well as consistency across batches [38,39].

The P-Gel-5% inserts formed a semi-interpenetrating network in this case where only one polymer phase, GelMA, was chemically crosslinked, and PVA was dispersed inside the GelMA matrix. Interestingly, PVA can also undergo supramolecular interactions (H-bonding) with GelMA chains, which contributes to the higher structural stability of the P-Gel-5% inserts. Reports suggest that the interpenetrating network hydrogels align with the requirements of extrusion-based 3D printing [40,41,42]. However, vat polymerization-based techniques offer higher geometrical complexity and higher shape fidelity [27]. While 3D printing involves a semi-interpenetrating network, it should be noted that the presence of PVA should not interfere with crosslinking of the acrylate species; thus, in this work, PVA was kept at a lower concentration of 5% *w*/*v* [43]. Also, the 3D printing conditions, such as the dye concentration and UV exposure time, were aptly selected, along with appropriate environmental conditions, including temperature (~37 °C) and humidity (~70% RH). It should be noted that despite the high water content of these inserts, they could be 3D-printed with a high shape fidelity characterized by flat surfaces and clean and sharp edges.

### 3.2. Mechanical Testing, Equilibrium Water Content (EWC), and Water Contact Angle

**Figure 3:**
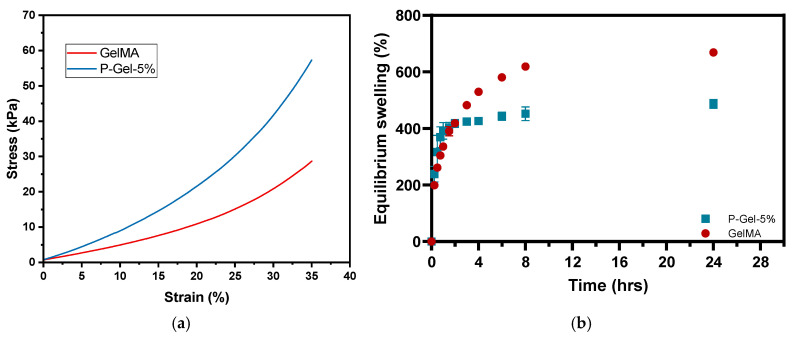
(**a**) Mechanical strength and (**b**) equilibrium swelling graphs for GelMA and P-Gel-5% samples.

The data from mechanical testing in compression mode (Figure 3a) indicate the physically stronger nature of the P-Gel-5% hydrogels compared to GelMA hydrogels. This could be attributed to the presence of PVA crystalline domains in the P-Gel-5% semi-interpenetrating network [44]. The addition of PVA to GelMA leads to the formation of hydrogen bonds and small ordered regions called crystalline domains dispersed inside the GelMA matrix. The PVA crystalline domains can be described as a layered structure held together by hydroxyl bonds along with weak van der Waals forces operating between two layers [45]. Higher amounts of PVA chains lead to the formation of more hydrogen bonds and crystalline regions, forming a relatively stiffer network [46].

Additionally, the supramolecular interactions (H-bonding) between the -OH/-NH_2_ groups of PVA and GelMA, respectively, also contribute to the higher mechanical strength and lower equilibrium swelling of the P-Gel-5% hydrogels. These supramolecular interactions as inferred from the scanning electron microscopy images of P-Gel-5% hydrogels, where P-Gel-5% forms a semi-interpenetrating network [36]. As previously mentioned, during the freezing step in the lyophilization process, thermally induced phase separation occurs between the solvent phase and the polymer phase, creating a polymeric network [47]. GelMA hydrogels display a larger pore size due to the presence of a lower polymeric phase in GelMA hydrogels (10% GelMA) compared to the P-Gel-5% hydrogels (10% GelMA and 5% PVA). Thus, the presence of a lower polymeric phase in GelMA hydrogels allows it to hold more water to undergo ice crystal formation during the freezing step of lyophilization. The PVA molecules might penetrate and partially fill the free voids between GelMA chains, thus forming smaller-sized pores between the crosslinked GelMA chains, thus increasing the crosslinking density. The increased crosslinked polymer fraction gives more resistance to the compressive force and, hence, higher mechanical strength to the P-Gel-5% hydrogels [48].

**Figure 4:**
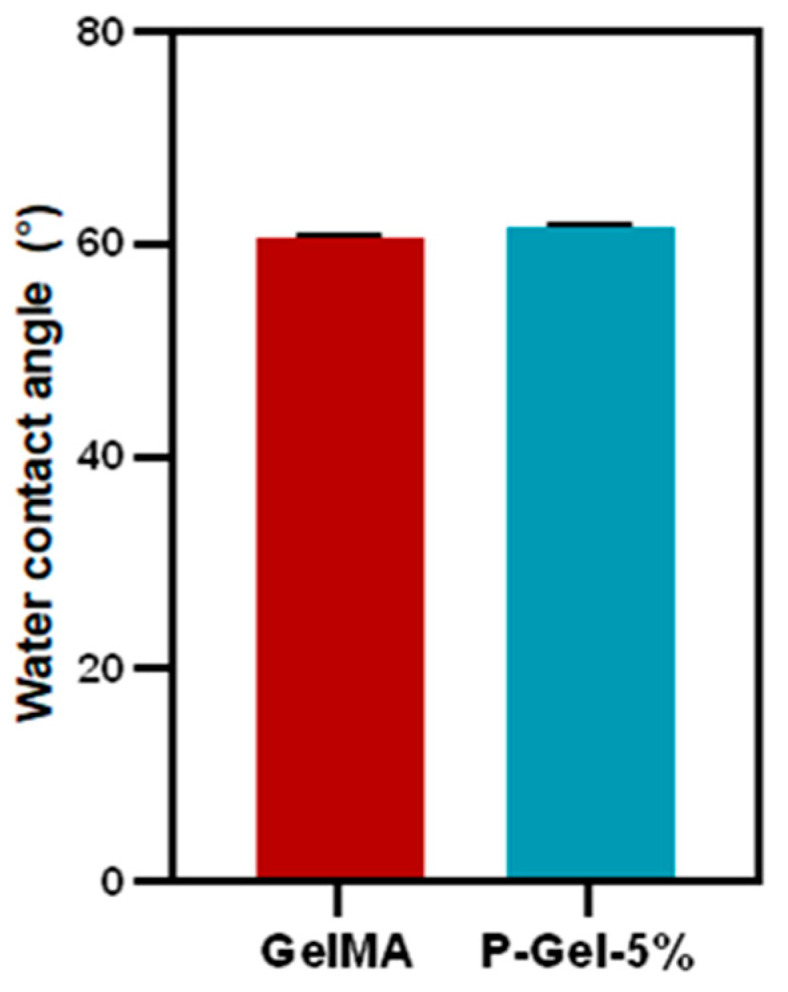
Water contact angle for GelMA and P-Gel-5% conjunctival inserts.

Reportedly, the molecular chains of gelatin can extend more easily compared to the PVA polymer chains [49]. This is further corroborated by the results from the equilibrium swelling curves for GelMA and P-Gel-5% hydrogels, as shown in Figure 3b. From the swelling curves, it can be inferred that there is a decrease in the degree of swelling in P-Gel-5% samples compared to GelMA hydrogels. Previous studies suggest that higher swelling leads to higher degradation and lower mechanical strength, which also aligns with our results [49]. The interplay between degradation rate and mechanical properties of GelMA/PVA hydrogels could be used to fabricate conjunctival inserts with desired functionalities. For instance, if the degradation rate is too fast, it would lead to the mechanical failure of these inserts, impacting the drug release profiles. Moreover, the relative amount of crystalline and amorphous phases of PVA present is a key determinant of the macroscopic properties of the P-Gel-5% hydrogels. An increase in the crystalline fraction of PVA can cause a decrease in the water permeability of the P-Gel hydrogels compared to GelMA [46,47,50,51]. Figure 4 shows the water contact angles of the GelMA and P-Gel-5% samples. The water contact angle of GelMA samples was 60.6°, and the P-Gel-5% sample’s angle was 61.5°, indicating their hydrophilic nature. These results were not significantly different from each other (*p* = 0.1268).

### 3.3. PVA Release from P-Gel-5% Inserts in the Presence of MMP9

**Figure 5:**
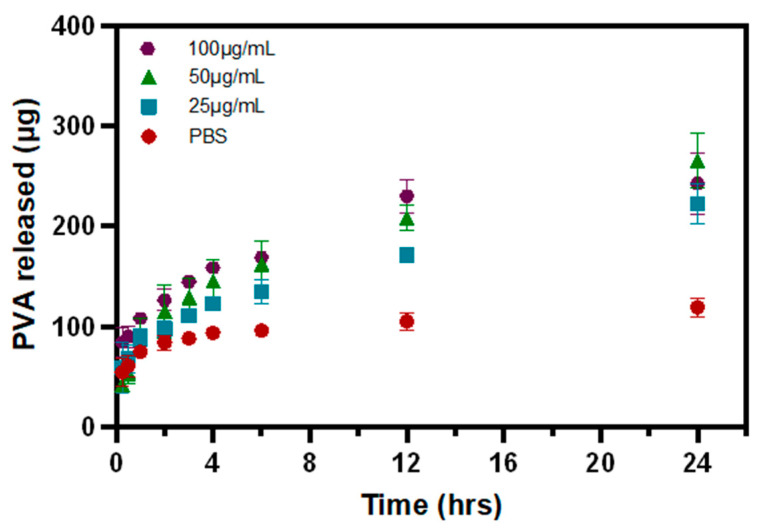
PVA release curves of 3D-printed P-Gel-5% conjunctival inserts in the presence of varying concentrations of MMP9 enzyme.

The PVA release curves depict the PVA released from the 3D-printed ocular inserts (Figure 5) in the presence of different concentrations of MMP9 enzymes (25 µg/mL, 50 µg/mL, and 100 µg/mL). About 119.5 ± 9.4 µg of PVA was released from P-Gel-5% inserts at the end of 24 h without the presence of MMP9. In the presence of the MMP9 enzyme, about 222.7 ± 20.3 µg, 265.5 ± 27.1 µg, and 242.7 ± 30.4 µg of PVA were released when exposed to 25 µg/mL, 50 µg/mL, and 100 µg/mL of MMP9 at the end of 24 h (Figure 5). This increased amount of PVA release can be attributed to faster disintegration of the semi-interpenetrating network in the presence of elevated enzyme concentrations, thus favoring higher release of PVA.

### 3.4. Biodegradation of P-Gel-5% Inserts in the Presence of MMP9

**Figure 6:**
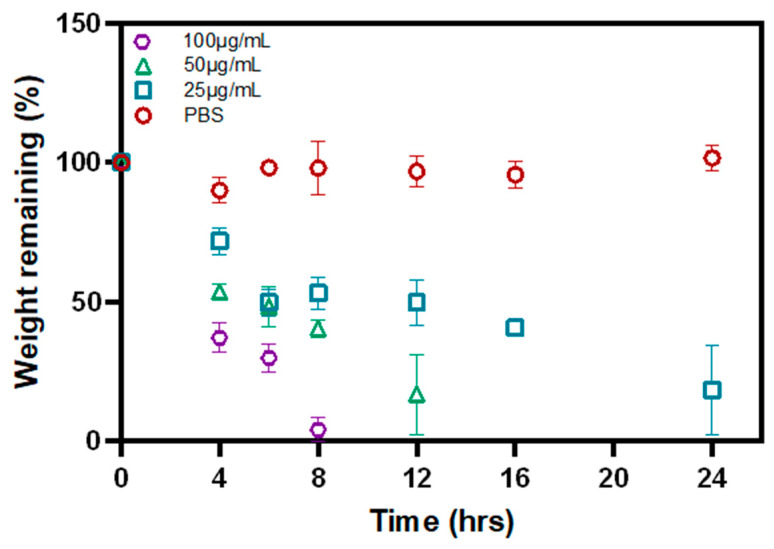
Biodegradation profiles of 3D-printed conjunctival inserts in the presence of varying concentrations of MMP9 enzyme.

Figure 6 shows the biodegradation profile of the P-Gel-5% conjunctival inserts in the presence of MMP9 enzyme. About 96.02%, 83.36%, and 81.72% of the P-Gel-5% inserts were degraded at the end of 8 h, 12 h, and 24 h in the presence of 100, 50, and 25 µg/mL of MMP9, respectively. These results suggest that enzyme concentration play a significant role in MMP-mediated degradation of GelMA hydrogels. Different MMPs have varying affinities for GelMA and degrade it at different rates; however, the degradation rate is a function of MMP9 enzyme concentration. Moreover, the concentration of specific MMPs in the surrounding environment influences the degradation kinetics.

**Figure 7:**
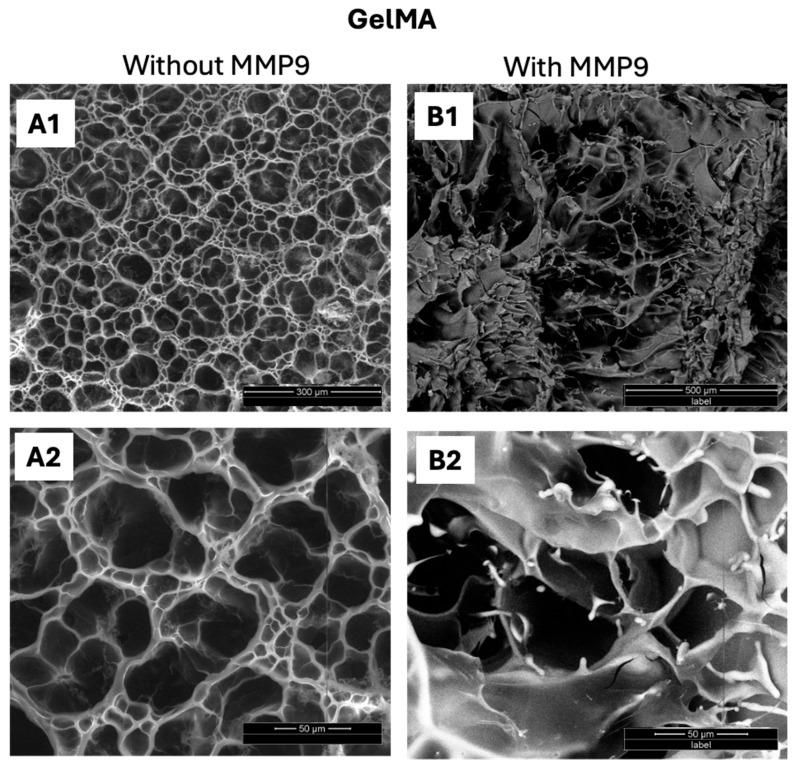
Morphological changes in GelMA inserts (**A1**,**A2**) before and (**B1**,**B2**) after exposure to 50 µg/mL MMP9 enzyme for 24 h.

No degradation was observed in the control groups incubated in PBS, at the end of the study duration of 24 h (Figure 7(A1,A2)). As previously stated, GelMA degrades in the presence of collagenases or MMPs, and these enzymes are found on the ocular surface. [7] MMP levels are elevated in certain ocular conditions such as corneal wound healing, ulceration, and uveitis, and they show collagenolytic activity [52]. Research shows that MMP-9 is elevated in the tears of dry eye patients [7,30]. The gelatinase MMP9 enzyme cleaves collagen chains α1(V) and α(XI) between Gly-439 and Val-440 and the α2(V) chain between Gly-445 and Leu-446 [31,32]. As degradation of gelatin chains occurs in the presence of MMPs [53], the mechanical properties of GelMA-based conjunctival inserts are also impacted by the MMP-mediated degradation process. The degradation profile of GelMA is affected by similar parameters that also influence the stiffness of the hydrogel, such as the degree of substitution [54], the concentration of photoinitiator used, and the intensity and time of UV exposure during the photocrosslinking process [55]. As the enzymes move through the polymer matrix, they break down crosslinks, effectively reducing the crosslink density (Figure 7(B1,B2)). This reduction leads to an expansion of the polymer mesh. Eventually, the degradation process reaches a critical point known as the reverse gelation point. At the reverse gelation point, the polymer undergoes a drastic shift in its structural integrity. The connectivity of the network declines sharply, causing a transition from a gel-like state to a more fluid-like state [56].

**Figure 8:**
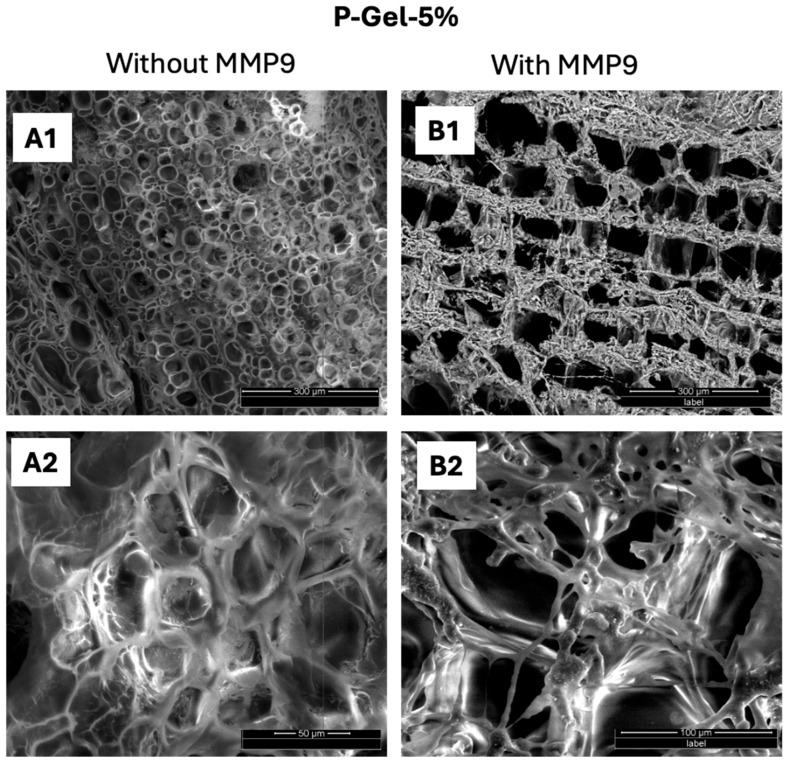
Morphological changes in P-Gel-5% inserts (**A1**,**A2**) before and (**B1**,**B2**) after exposure to 100 µg/mL MMP9 enzyme for 24 h.

Interestingly, upon the addition of PVA to GelMA, hydrogen bonds are formed between PVA and GelMA [36]. Moreover, owing to the inherent crystalline nature of PVA, small ordered regions called crystalline domains are scattered inside the GelMA matrix, which leads to the formation of a relatively stiffer network (Figure 8(A1,A2)) [46]. This is further supported by previous reports, which suggest that the reduced pore size in highly crosslinked GelMA-based soft hydrogels may create a membrane-like structure that is difficult for the MMP enzymes to penetrate through. Owing to the mechanically robust nature of P-Gel-5%, increased crosslinking density, and narrower pore size, the time needed for enzymatic cleavage is longer [26]. This could be the plausible explanation for the slower biodegradation rate of P-Gel-5% in the presence of MMP9 (Figure 8(B1,B2)) compared to GelMA alone, as shown in our previous works [57].

**Figure 9:**
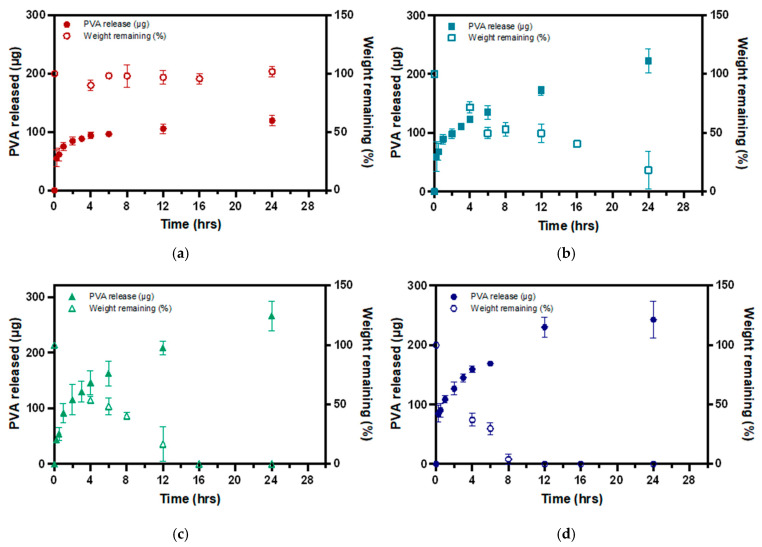
Correlation between the biodegradation profiles and PVA release curves of 3D-printed conjunctival inserts in the presence of varying concentrations of MMP9 enzyme (**a**) control, (**b**) 25 µg/mL, (**c**) 50 µg/mL, and (**d**) 100 µg/mL.

The incorporation of PVA into the GelMA network facilitates the formation of hydrogen bonds, leading to the development of crystalline domains that are dispersed within the GelMA matrix [36]. This suggests that the release of PVA is dependent on the rate of degradation of the GelMA network because the environmental conditions, such as incubation temperature and pH, are kept constant. Therefore, disruption of this H-bonding, and, hence, PVA release, is mediated by MMP9 enzymes acting on the GelMA network structure. As discussed earlier, the rate of GelMA degradation is a function of MMP9 enzyme concentration; therefore, higher enzyme concentrations display faster degradation and, hence, the faster release of PVA (Figure 9d). As the enzyme concentration decreases to 50 µg/mL (Figure 9c) and 25 µg/mL (Figure 9b), the rate of degradation becomes slower, and, consequently, the rate at which the PVA-GelMA supramolecular interactions (H-bonding) are disrupted is slower. This is evident from the slope of the PVA release curves in Figure 9. However, in control samples where no enzyme was present, no degradation of the P-Gel-5% insert was observed (Figure 9a), and the PVA release from these samples was relatively low and at a slower rate, as observed from the slope of these curves in the first few hours of release.

### 3.5. Release Kinetics in the Presence of MMP9 Enzymes

**Figure 10:**
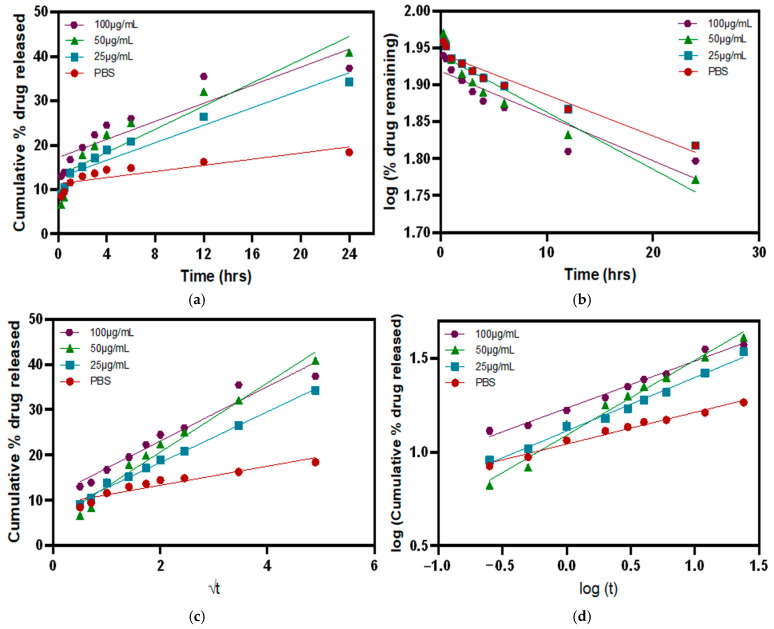
Modeling of the PVA release data on well-known mathematical models: (**a**) zero order, (**b**) first order, (**c**) Higuchi’s model, and (**d**) Korsmeyer–Peppas model.

To study the drug release kinetics and how different factors affect the dissolution velocity and behaviors, various mathematical equations describing the release dependence on time were used. The drug release was evaluated through four main release kinetic models: zero-order, first-order, Higuchi model, and Korsmeyer–Peppas model (Figure 10). To evaluate the best-fit model for the release profiles in our study, the goodness of fit for each model or the coefficient of determination (*R*^2^) was calculated using GraphPad Prism 10 (Table 3). The release profiles of the P-Gel-5% containing hydrogels in the presence of different concentrations of MMP9 showed the highest linearity with the Korsmeyer–Peppas model, as shown in Table 3. The release exponent (*n*) for the Korsmeyer–Peppas model was calculated from the slope of the graph between log(M*_t_*/M_∞_) versus log *t*. The *n*-values indicate that these systems follow Fickian diffusion. In the Fickian model (Case I), the drug release is governed by diffusion, and the rate of diffusion is greater than the process of polymeric chain relaxation. Equilibrium of absorption in the surface exposed to the polymeric system takes place rapidly, leading to conditions of time-dependent links. The kinetics of this phenomenon are characterized by a diffusivity [58].

## 4. Conclusions

In summary, this work highlights the versatile 3D-printing abilities of GelMA to design conjunctival inserts. The results from this study demonstrate the mechanism of degradation of GelMA/PVA-based conjunctival inserts by MMP9 enzymes and its correlation with the release of PVA, a well-known wetting agent for the treatment of dry eyes. Interestingly, these MMP9 enzymes are also found on the ocular surface and are elevated in ocular conditions such as dry eye disease. These elevated levels can be utilized as a feedback mechanism for regulating the degradation rate of PVA from the inserts. The degradation rate of GelMA/PVA is directly correlated with the concentration of the MMP9 enzyme. As the enzyme moves through the polymer matrix, it breaks down crosslinks, reducing the overall crosslink density. Consequently, higher enzyme concentrations accelerate the disruption of PVA-GelMA supramolecular interactions (H-bonding), leading to a faster release of PVA. The release profiles of the P-Gel-5% containing hydrogels in the presence of different concentrations of MMP9 showed the highest linearity with the Korsmeyer–Peppas model. These inserts hold promise as successful drug-delivery devices for the treatment of dry eyes. However, before these inserts can be considered as a commercial product, a complete set of in vitro and in vivo studies needs to be evaluated. In the future, these inserts could also be explored to deliver a variety of therapeutic molecules of interest to treat various ocular conditions.

## Figures and Tables

**Table 1:** Composition of GelMA and P-Gel-5% ink.

	GelMA	PVA	LAP	Tartrazine
GelMA ink	10	0	0.6	0.3
P-Gel-5% ink	10	5	0.6	0.3

(values reported as % *w*/*v*).

**Table 2:** Curve fitting of various models for drug release kinetics.

	Zero Order	First Order	Higuchi Model	Korsmeyer-Peppas Model
Equation	C_t_ = C_o_ + K_o_*t*	logC_t_ = logC_o_ − (K_1_/2.303)*t*	M*_t_*/M_∞_ = K*_h_t*^1/2^	log(M*_t_*/M_∞_) = logK*_kp_* + *n* log *t*
Graph	cumulative drug release vs. time	log cumulative % drug remaining vs. time	cumulative % drug release vs. √t	log cumulative % drug release vs. log *t*

**Table 3:** The coefficient of determination (*R*^2^) values of PVA release from P-Gel-5% in PBS and MMP9 (25 µg/mL, 50 µg/mL, and 100 µg/mL) calculated by using different kinetic models.

Kinetic Model	PBS	25 µg/mL	50 µg/mL	100 µg/mL
Zero-order	0.7143	0.9160	0.8490	0.8135
First order	0.9420	0.9420	0.8983	0.8399
Higuchi	0.8910	0.9947	0.9734	0.9528
Korsmeyer–Peppas	0.9785	0.9896	0.9812	0.9809
Diffusion exponent (*n*-value) *	0.1685	0.2852	0.3995	0.2518

* Calculated by the linear regression of Korsmeyer–Peppas equation of log(M*_t_*/M_∞_) versus log *t*.

## Data Availability

Data is contained within the article.
